# Disseminated fungal infection in a patient receiving zanubrutinib

**DOI:** 10.1002/ccr3.7564

**Published:** 2023-06-13

**Authors:** Madalyn Walsh, Alice Xu, Zuhair Ballas, Rolando Sanchez

**Affiliations:** ^1^ Department of Internal Medicine University of Iowa Hospitals & Clinics Iowa City Iowa USA; ^2^ Division of Immunology University of Iowa Hospitals & Clinics Iowa City Iowa USA; ^3^ Division of Pulmonary, Critical Care and Occupational Medicine University of Iowa Hospitals & Clinics Iowa City Iowa USA; ^4^ Pulmonary Medicine, VA Iowa City Health Care Virginia Medical Center Iowa City Iowa USA

**Keywords:** aspergillus, ibrutinib, lymphoma, zanubrutinib

## Abstract

We report a case of fatal disseminated aspergillosis in the setting of administration of zanubrutinib, a second‐generation Bruton's tyrosine kinase inhibitor thought to have a lower rate of immunosuppression‐related side effects.

## Graphical abstract

MRI of the brain and CT angiogram of the chest revealing several lesions suggestive of widespread fungal disease.

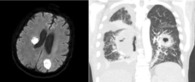

## 1 INTRODUCTION

Zanubrutinib is a second‐generation Bruton's tyrosine kinase inhibitor (BTKi) often used to treat B cell malignancies such as marginal zone lymphoma. BTKis belong to a protein tyrosine kinase family that plays an essential role in the proliferation, differentiation, and survival of B cells. Significant side effects of BTKis include infection, diarrhea, skin rash, and atrial fibrillation, which appear to be related to their broad inhibitory activity across the TEC family and other tyrosine kinases. Invasive fungal infections have been previously described with ibrutinib (a first‐generation BTKi) but seldom with second‐generation BTKis such as zanubrutinib.

## 2 CASE PRESENTATION

A 76‐year‐old male with history of marginal zone lymphoma diagnosed after presenting with diffuse bulky adenopathy and subsequent excisional biopsy. Additional medical history included benign prostatic hypertrophy, hyperlipidemia, hypertension, gout, and atrial flutter while receiving ibrutinib. Medications included allopurinol, apixaban, atorvastatin, metoprolol tartrate, and tamsulosin. He had a 10 pack‐year history of tobacco use and endorsed drinking one standard alcoholic beverage daily. Initial treatment for his lymphoma included three cycles of rituximab–bendamustine (R‐Benda), with 90% reduction in lymphadenopathy on imaging surveillance. Several years later, he developed malignant recurrence manifested by diffuse lymphadenopathy and was restarted on R‐Benda. Due to the progression of his disease while on this therapy, he was started on ibrutinib (a first‐generation BTKi) as salvage therapy. However, the patient developed diarrhea and intermittent episodes of atrial flutter requiring dose reduction and subsequently the discontinuation of the drug. Therapy was then switched to zanubrutinib (a second‐generation BTKi) with good clinical response.

**FIGURE 1:**
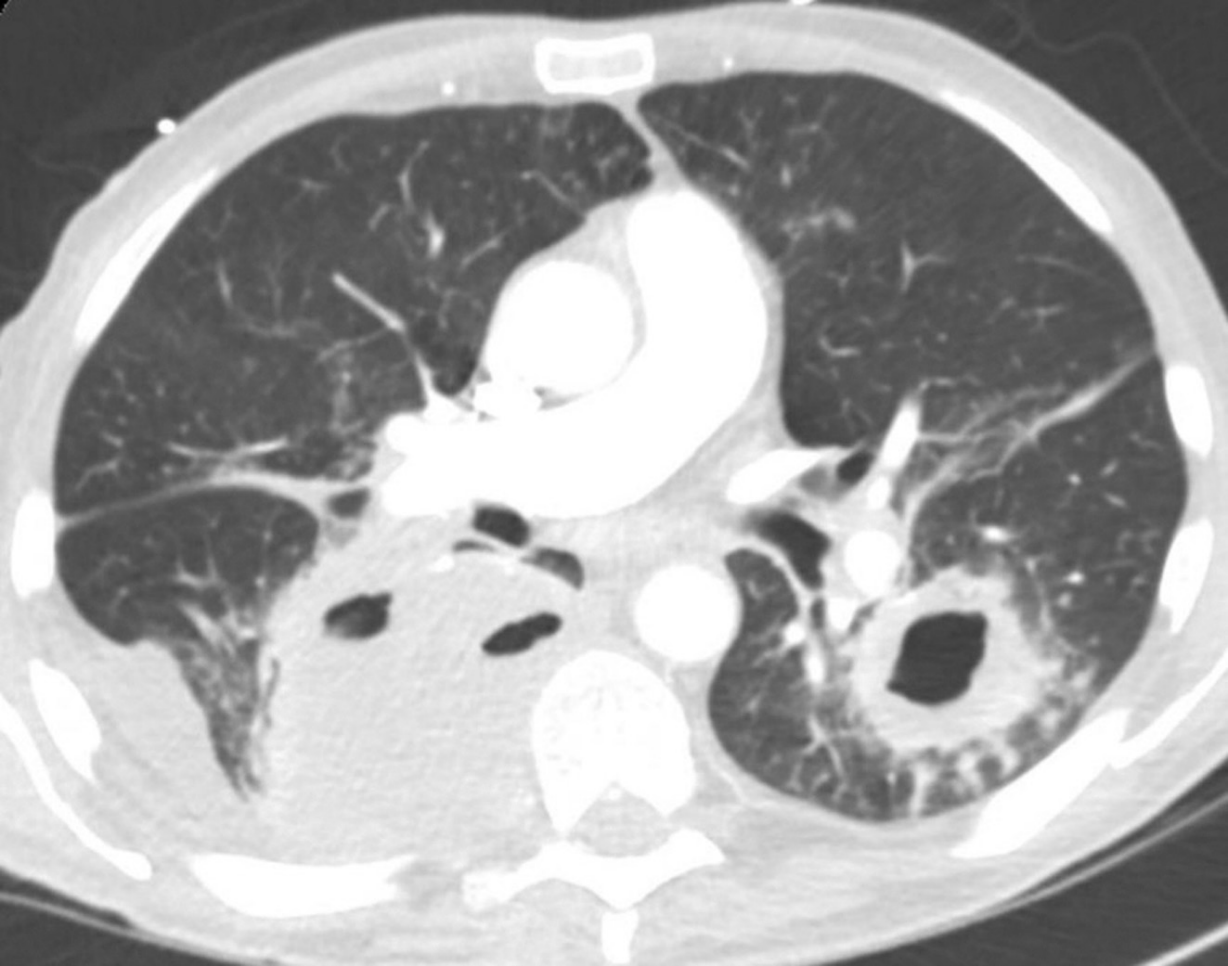
CT angiogram of the chest in transverse view revealing large bilateral lung abscesses.

After 6 months of therapy with zanubrutinib, the patient developed chest pain and dyspnea. Chest x‐ray was performed revealing bilateral alveolar opacities. Antibiotic therapy was prescribed for presumptive bacterial pneumonia without improvement of symptoms. CT scan of the chest redemonstrated bilateral alveolar opacities. The patient underwent a CT‐guided needle biopsy of the lung opacities revealing pathological signs of organizing inflammation. The patient was diagnosed with cryptogenic organizing pneumonia and treated with a moderate dose of prednisone. CT angiogram of the chest done months later to rule out pulmonary embolism in the setting of respiratory distress revealed worsening of alveolar opacities with bilateral lung abscess formation and a new right sided pleural effusion (Figure 1). The patient developed worsening dyspnea, weight loss, and decline in functional status requiring admission to the intensive care unit. Zanubrutinib was held on admission and steroid therapy tapered quickly due to concerns for an underlying opportunistic infection.

Laboratory investigations revealed leukopenia with white blood cell count of 4.2 K/μL, a new neutropenia with absolute neutrophil count of 357 cells/μL, anemia with hemoglobin of 9.8 g/dL (*N* 14–18 g/dL), thrombocytopenia with platelet count of 34 K/μL (*N* 150–450 K/μL), IgA 47 mg/dL (*N* 70–400 mg/dL), IgM 19 mg/dL (*N* 40–230 mg/dL), IgG 426 mg/dL (*N* 700–1600 mg/dL), and completely absent natural killer (NK) cell cytotoxic activity. Histoplasma antigen in both serum and urine was negative. Coccidioides antibody was negative. Human immunodeficiency virus (HIV) was negative. Serum galactomannan was positive at 9.56 (*N* < 0.5 index). Blood and urine cultures were obtained and were negative.

A right‐side thoracentesis was performed and revealed an uncomplicated exudative effusion with lymphocyte predominance. A bronchoscopy was performed with bronchoalveolar lavage (BAL) and transbronchial lung biopsies. The pathology examination showed abundant necrotic inflammation with broad ribboning hyphae consistent with invasive fungal infection. Pathology from left lower lobe biopsy was significant for hyphal elements with septations present, concerning for aspergillus or mucormycosis. Cases published pertaining to invasive fungal infections in patients receiving BTKis had aspergillus as the most common underlying organism. Based on this data and the marked elevation of serum galactomannan, a decision was made to treat for the patient for disseminated aspergillosis.

**FIGURE 2:**
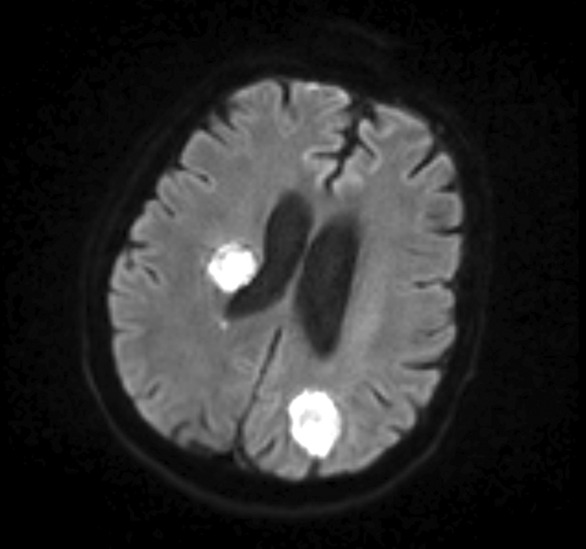
MRI of the brain revealing several intracranial ring‐enhancing lesions with surrounding edema suggestive of fungal abscess.

The patient had initially received conservative antibiotic coverage with vancomycin and cefepime which were discontinued after disseminated fungal infection was confirmed. He was started on voriconazole as well as micafungin for synergistic effect. The patient's condition continued to deteriorate, and he developed worsening encephalopathy with left side hemiparesis. A diffusion‐weighted imaging MRI of the brain was performed and revealed numerous lesions in the cerebellum and cerebrum bilaterally with surrounding vasogenic edema without midline shift or obstructive hydrocephalus (Figure 2).

Dexamethasone was initiated to decrease edema and was weaned while preserving mentation. Intravenous immunoglobulin (IVIG) therapy was also administered due to low levels of IgG and for immunomodulation at a dose of 1 g/kg/day for 2 days. Despite aggressive measures, the patient's mentation and respiratory status continued to decline. His family elected to pursue a comfort‐based approach to care, and he died several days later. Final culture data from the BAL confirmed the diagnosis of disseminated aspergillosis.

## 3 DISCUSSION

Bruton's tyrosine kinase belongs to a sub‐family of non‐receptor protein tyrosine kinases (TEC kinases) and plays an essential role in the proliferation, differentiation, and survival of B cells. BTK also plays a role in the function of innate cells and T cells and mediates Toll‐like receptor signaling and NLRP3 inflammasome activation.^1^ BTKis have revolutionized landscape therapy of B cell malignancies such as chronic lymphocytic leukemia, diffuse large B cell lymphoma, mantle cell lymphoma, and Waldenstrom macroglobulinemia.

Ibrutinib was the first‐generation BTKi to be approved for the treatment of B cell malignancies. It binds irreversibly to BTK. Although it was generally well tolerated with rapid and durable response in clinical studies, the rate of discontinuation of this drug in community practice due to side effects is reportedly as high as 49%. The side effects of ibrutinib, including diarrhea, skin rash, infections, bleeding, atrial fibrillation, are thought to be associated to off binding of other protein tyrosine kinases. The next generation of BTKi, like zanubrutinib, have improved BTK binding profiles and selectivity, and were developed to decrease the rate of side effects observed with ibrutinib.^2^, ^3^ Serious side effects, such as disseminated fungal infections, are now being described with the next‐generation BTKis.

Disseminated fungal infections have been frequently described in patients treated with ibrutinib, with aspergillus being the most common pathogen. The underlying mechanism of BTKis causing impairment of the immune response to fungal infection is not well understood, but it appears to be related to not only B cell dysfunction but also macrophage and neutrophil dysfunction. Mutations in the human BTK gene results in the development of X‐linked agammaglobulinemia. This disorder is characterized by a primary humoral immunodeficiency where B cell development is arrested with subsequent almost total lack of immunoglobulin production. While patients with X‐linked agammaglobulinemia have increased risk of opportunistic infections, disseminated fungal infections are rarely seen in this population. Studies suggest that on and off target inhibition of tyrosine kinases by BTKis mediate the dysfunction of neutrophils, macrophages, T cells, and platelets as well as abnormal Toll‐like receptors which may play a role in the impairment of fungal immune response.^4^, ^5^

In the presented case, the patient had several factors that predisposed him to a higher risk of disseminated aspergillus infection. The first factor was that he was treated with ibrutinib for 2 years prior to transition to zanubrutinib, exposing him to potential immune dysfunction. Second, he was diagnosed with presumptive cryptogenic organizing pneumonia on initial presentation and was started on a prolonged steroid course. He may have already been infected with aspergillosis pneumonia at this time. The steroids likely caused further suppression of neutrophil function in the setting of zanubrutinib use. In a small trial of primary central nervous system lymphoma treated with a combination of ibrutinib and steroids, up to 39% of patients developed invasive aspergillosis.^6^

Zanubrutinib is a newer BTKi that is advertised as having greater specificity for the BTK receptor compared to the prior formulations.^7^ Compared to ibrutinib, there is reduced affinity to off‐target receptors such as epidermal growth factor receptor (EGFR), interleukin 2‐inducible T cell kinase (ITK), and Janus kinase 3 (JAK3).^2^ These receptors ensure appropriate neutrophil and macrophage function, thus improving the innate immune response against fungal infections in those treated with novel BTKi. However, there are currently limited studies comparing the affinity of BTK receptors on innate immune cells, as BTK is also involved in neutrophil recruitment and activation along with macrophage phagocytosis.^7^ Given the high mortality rate of these disseminated fungal infections, a better understanding of the underlying mechanisms of BTK impairing the immune system response to fungal infections would likely impact clinical decisions regarding its use.

In review of published cases of infections in the setting of ibrutinib use, multiple factors seem to impact the patient's innate immune response. A majority of the patients were treated for CLL, a condition that causes immunosuppression in itself. Most patients had other pre‐disposing risk factors such as prior immunotherapy and chemotherapy, steroid use, and neutropenia. A few patients had no other factors other than initiation of ibrutinib. Thus far, there has been one reported case of fungal infection with cryptococcus with use of zanubrutinib complicated by neutropenia and prior rituximab use with persistent cytopenia.

## 4 CONCLUSION

Here, we present a case of disseminated aspergillus infection in the setting of zanubrutinib use in a patient with marginal zone lymphoma. His clinical course was likely impacted by a prolonged steroid course which resulted in dysfunction of his innate immune system and neutropenia in the setting of BTKi administration. Due to the limited time that this medication has been on the market and the limited number of published cases pertaining to adverse events, it is difficult to assess whether or not the greater propensity of neutropenia in zanubrutinib increases the likelihood of fungal infection. Zanubrutinib may also play a role in decreased neutrophil function in the remaining cells present. We expect that there will be greater understanding as additional cases are presented.

## AUTHOR CONTRIBUTIONS

**Madalyn Walsh:** Conceptualization; data curation; formal analysis; investigation; project administration; resources; supervision; writing – original draft; writing – review and editing. **Alice Xu:** Conceptualization; formal analysis; investigation; resources; writing – original draft; writing – review and editing. **Zuhair Ballas:** Investigation; supervision; writing – review and editing. **Rolando Sanchez:** Conceptualization; investigation; resources; supervision; writing – review and editing.

## FUNDING INFORMATION

Funding was received from the University of Iowa.

## CONFLICT OF INTEREST STATEMENT

The authors have no conflicts of interest to disclose.

## ETHICS STATEMENT

Ethical approval is not required by the Institutional Review Board for case report publication.

## CONSENT

Written informed consent was obtained from the patient to publish this report in accordance with the journal's patient consent policy.

## Data Availability

Data sharing is not applicable to this article as no new data were created or analyzed in this study.
